# Umbilical Cord-Derived CD362^+^ Mesenchymal Stromal Cells Attenuate Polymicrobial Sepsis Induced by Caecal Ligation and Puncture

**DOI:** 10.3390/ijms21218270

**Published:** 2020-11-04

**Authors:** Hector Gonzalez, Colm Keane, Claire H. Masterson, Shahd Horie, Stephen J. Elliman, Brendan D. Higgins, Michael Scully, John G. Laffey, Daniel O’Toole

**Affiliations:** 1 Anaesthesia, School of Medicine, National University of Ireland, Galway H91 TK33, Ireland; h.gonzalez2@nuigalway.ie (H.G.); colmpkeane@hotmail.com (C.K.); claire.masterson@nuigalway.ie (C.H.M.); shahd.horie@nuigalway.ie (S.H.); michael.scully@nuigalway.ie (M.S.); 2 Regenerative Medicine Institute, National University of Ireland, Galway H91 TK33, Ireland; 3 Orbsen Therapeutics Ltd., Galway H91 DET7, Ireland; steve.elliman@orbsentherapeutics.com; 4 Physiology, School of Medicine, National University of Ireland, Galway H91 TK33, Ireland; brendan.higgins@nuigalway.ie

**Keywords:** mesenchymal stem cell, sepsis, inflammation

## Abstract

Mesenchymal stromal cells (MSCs) have a multimodal, immunomodulatory mechanism of action and are now in clinical trials for single organ and systemic sepsis. However, a number of practicalities around source, homogeneity and therapeutic window remain to be determined. Here, we utilised conditioned medium from CD362^+^-sorted umbilical cord-human MSCs (UC-hMSCs) for a series of in vitro anti-inflammatory assays and the cryopreserved MSCs themselves in a severe (Series 1) or moderate (Series 2+3) caecal ligation and puncture (CLP) rodent model. Surviving animals were assessed at 48 h post injury induction. MSCs improved human lung, colonic and kidney epithelial cell survival following cytokine activation. In severe systemic sepsis, MSCs administered at 30 min enhanced survival (Series 1), and reduced organ bacterial load. In moderate systemic sepsis (Series 2), MSCs were ineffective when delivered immediately or 24 h later. Of importance, MSCs delivered 4 h post induction of moderate sepsis (Series 3) were effective, improving serum lactate, enhancing bacterial clearance from tissues, reducing pro-inflammatory cytokine concentrations and increasing antimicrobial peptides in serum. While demonstrating benefit and immunomodulation in systemic sepsis, therapeutic efficacy may be limited to a specific point of disease onset, and repeat dosing, MSC enhancement or other contingencies may be necessary.

## 1. Introduction

Sepsis is a life-threatening syndrome caused by bacterial infection [1] in which patients develop an inflammatory response to a pathogen, producing shock and organ damage that can lead to death with an overall mortality rate of 40% [2]. The most common causes of sepsis are infections in the lungs, abdominal cavity, urinary tract and soft tissue [2]. The inflammatory reaction is a result of the generation of diverse pro-inflammatory molecules including tumour necrosis factor (TNF)-α, interleukin (IL)-1β, IL-2, IL-6, IL-8 and interferon (IFN)-γ. This “cytokine storm” is responsible for early sepsis-related multiple organ failure and death [3]. Mesenchymal stromal cells (MSCs) are a promising therapeutic strategy for the treatment of sepsis due to their reported immunomodulatory properties [4] and have been shown to have a multimodal mechanism of action involving cytokine and other factor production, secretion of extracellular vesicles and even cell–cell contact-dependent processes [5,6,7]. Previous studies have shown the efficacy of these cells to improve the outcome of sepsis of different aetiologies [8,9]. Our previous research has shown that bone-marrow-derived MSCs enhance the resolution of *E. coli* pneumonic sepsis [10] and ventilator-induced lung injury (VILI) animal models [11]. There are also ongoing clinical trials investigating the beneficial effect of MSCs in sepsis patients (NCT03369275, NCT02883803).

Despite the promise of MSC therapy, there are still challenges to creating MSC doses at a scale to facilitate large clinical studies. Current isolation of MSCs from various tissue sources is based on plastic adherence, characterisation by standard surface markers using flow cytometry and multilineage differentiation assays [12]. This process results in populations of cells with different differentiation capacities and development stages that can jeopardise the therapeutic effect and increase differences between batches. This can make it difficult to achieve the standards for advanced therapeutic medicinal products (ATMP) for clinical use [13]. CD362, also known as syndecan 2, is a surface marker found on a subpopulation of human mesenchymal stromal cells, and has been used to facilitate MSC isolation by fluorescence-activated cell sorting (FACS), streamlining the isolation process. The immunomodulatory activity of bone-marrow-derived CD362^+^ human MSCs in vitro and in vivo has been previously reported in a relevant model of *E. coli* pneumonia, decreasing pneumonia severity and improving recovery after ventilation injury [14]. Recently our laboratory has showed the efficacy of umbilical-cord-derived CD362^+^ human MSCs (UC-hMSCs) in the resolution of pneumonia and the maintenance of MSC immunomodulatory activity after cryopreservation of the cells [15]. In related bacterial infection and inflammation models, we have also demonstrated a paracrine effect, where delivery of MSC to distal compartments such as the peritoneum or conditioned medium derived from MSCs exerted similar antibacterial and therapeutically beneficial effects to the MSC itself administered systemically or directly to the site of injury [10,16,17].

Here, we wished to prove the therapeutic potential of UC-derived CD362^+^ hMSCs (trademark Orbcel-C^®^) in a polymicrobial systemic sepsis using a caecal ligation and puncture (CLP) model.

## 2. Results

### 2.1. CD362^+^ UC-MSCs Demonstrate Immunomodulatory, Proreparative, Antibacterial and Prosurvival Effects In Vitro

**Figure 1:**
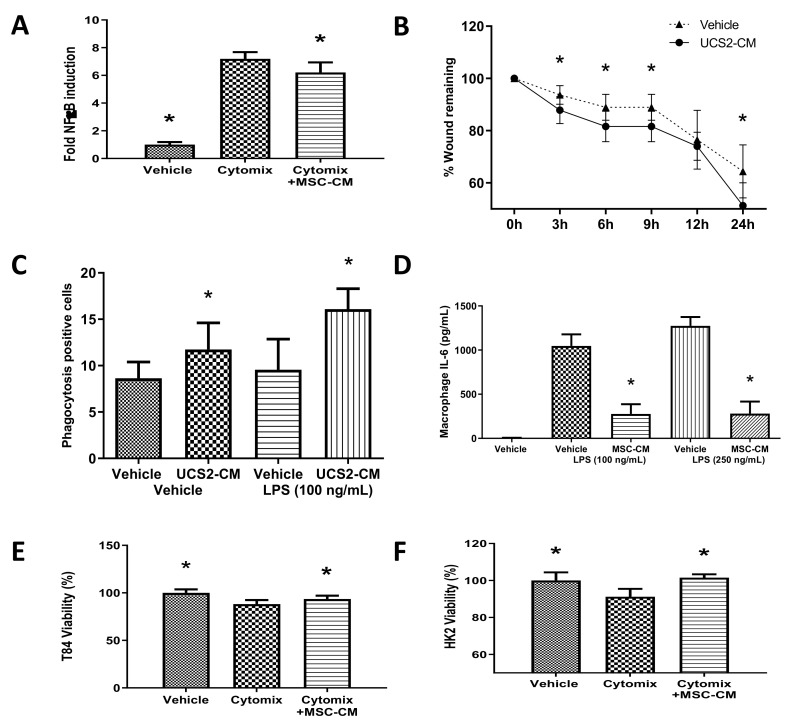
Anti-inflammatory capacity of CD362^+^ umbilical cord mesenchymal stromal cells conditioned medium (UC-MSC-CM) in vitro. CD362^+^ UC-MSC-CM decreased interleukin 1β (IL-1β)-induced activation of the nuclear factor kappa B (NF-κB) pathway (**A**) and enhanced wound closure (**B**) in pulmonary epithelial cells. CD362^+^ UC-MSC-CM increased the rate of phagocytosis in THP-1 macrophages (**C**) and reduced the production of IL-6 in peritoneal macrophages in response to lipopolysaccharide (LPS) (**D**). In other sepsis-relevant tissue cells, CD362^+^ UC-MSC-CM improved viability in kidney-derived HK2 (**E**) and gut-endothelial-derived T84 cells (**F**) after cytomix-induced injury. * Statistically significant (*p* < 0.05) with respect to cytomix group. CD362^+^ UC-MSCs reduce IL-6 peritoneal macrophage production after LPS stimulation (**D**). * Statistically significant (*p* < 0.05) with respect to cytomix (**A**,**E**,**F**), vehicle at (**B**) or vehicle at same LPS concentration (**C**,**D**). Columns represent mean (*n* = 6), error bars represent SD.

Rationale: to demonstrate the paracrine mechanism of action of the MSC. Conditioned media derived from CD362^+^ UC-MSC culture significantly attenuated cytomix-induced nuclear factor kappa B (NF-κB) activation in type II alveolar A549 cells compared with vehicle control (Figure 1A), indicating an anti-inflammatory effect in a sepsis-relevant tissue type. Conditioned medium also accelerated wound closure in lung epithelial cell monolayers (Figure 1B). Next, we sought to model the immundomodulatory effect of the MSC on the crucial macrophage response in the presence of a pathogen-derived stimulus. CD362^+^ UC-MSC-conditioned medium (CM) increased the rate of phagocytosis in THP-1-derived macrophages (Figure 1C), while the same CM reduced primary peritoneal macrophage secretion of the pro-inflammatory cytokine IL-6 (Figure 1D), both in the presence of *E.coli*-derived lipopolysaccharide (LPS). Finally, we sought to reflect chronic organ injury when extended exposure to an inflammatory environment causes cell death. CD362^+^ UC-MSC-CM improved cell viability in kidney-derived HK2 cells (Figure 1E) and gut-derived T84 cells (Figure 1F) after cytomix stimulation compared with vehicle control, suggesting a direct supportive effect of MSC in organ failure.

### 2.2. CD362^+^ hMSCs Improve Survival in Severe Systemic Sepsis

**Figure 2:**
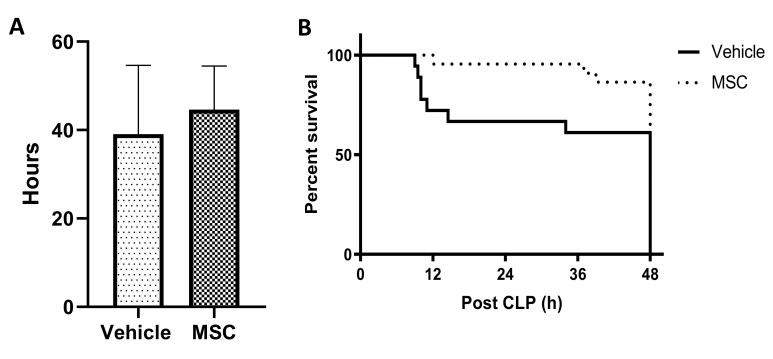
Survival after severe caecal ligation and puncture (CLP) injury and CD362^+^ UC-MSC administration. Administration of CD362^+^ UC-MSCs contemporaneous with severe systemic sepsis induction increased mean survival duration (**A**, *n* = 32/14) and total survival at the defined 48 h timepoint (**B**). Bars represent mean (*n* = 32/14), error bars represent SD.

Rationale: To determine the ability of the MSC to reduce mortality in severe sepsis. Administration of CD362^+^ UC-MSCs contemporaneously with induction of severe systemic sepsis improved mean survival duration (Figure 2A) and survival at 48 h (Figure 2B) compared to PBS controls. No mortality was observed in the moderate CLP injury protocol (Series 2 and Series 3).

### 2.3. CD362^+^ hMSCs Improve Hyperlactatemia and Bacterial Clearance at 4 h Administration Post Moderate CLP

**Figure 3:**
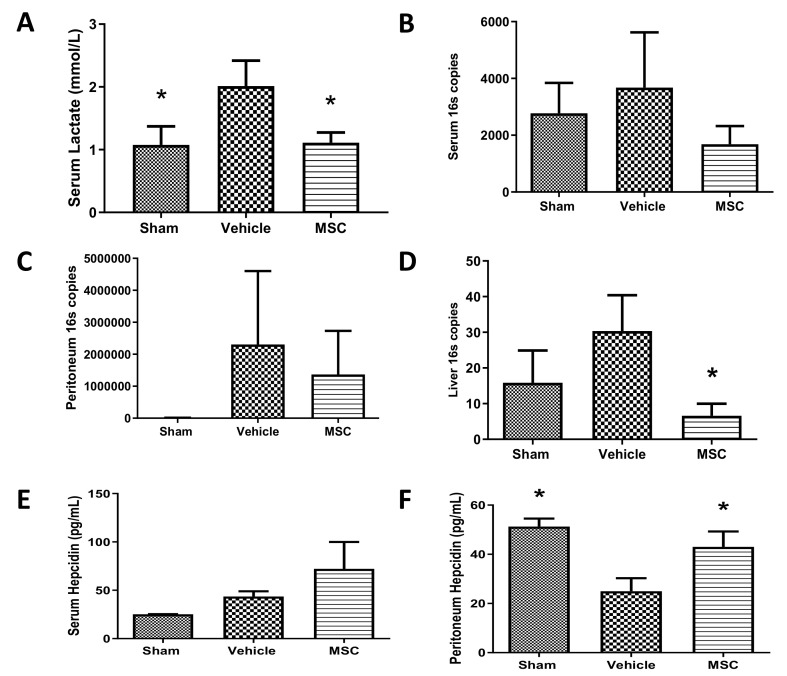
Reduced serum lactate and bacterial genome number and increased antimicrobial peptide after CD362^+^ UC-MSC administration at 4 h. Administration of CD362^+^ UC-MSCs 4 h after CLP induction reduced the lactate measured in serum 48 h after the CLP procedure compared with vehicle (**A**). Reduction of copies of the 16S fraction of the bacterial genome in serum after CD362^+^ UC-MSC administration. (**B**). Reduction of copies of 16S fraction of bacterial genome in peritoneal lavage serum after CD362^+^ UC-MSC administration (**C**). Reduction of copies of 16S fraction of bacterial genome in the liver after CD362^+^ UC-MSC administration (**D**). Increased concentration of the antimicrobial peptide hepcidin in serum after CD362^+^ UC-MSC treatment compared with vehicle control (**E**). Increased presence of the antimicrobial peptide hepcidin in peritoneal lavage in the CD362^+^ UC-MSC treated group compared with vehicle control (**F**). Bars represent mean (*n* = 3/8/8), error bars represent SD. * Statistically significant (*p* < 0.05) with respect to vehicle group.

Rationale: To determine if MSCs improve biomarkers and infection status in moderate sepsis. Administration of CD362^+^ UC-MSCs 24 h after of CLP induction failed to reduce lactate or cytokine marker levels resulting from CLP-induced sepsis (Supplemental File S1) but administration of CD362^+^ UC-MSCs 4 h after CLP induction significantly reduced lactate measured in serum (Figure 3A), bacterial presence in serum (Figure 3B), peritoneal lavage (Figure 3C) and liver (Figure 3D) measured at 48 h after CLP by QPCR. Administration of CD362^+^ UC-MSCs also increased the concentrations of antibacterial peptide hepcidin in serum and peritoneal lavage (Figure 3E,F).

### 2.4. CD362^+^ UC-MSC Administration Reduces Organ Viable Bacterial Load in Severe Systemic Sepsis

**Figure 4:**
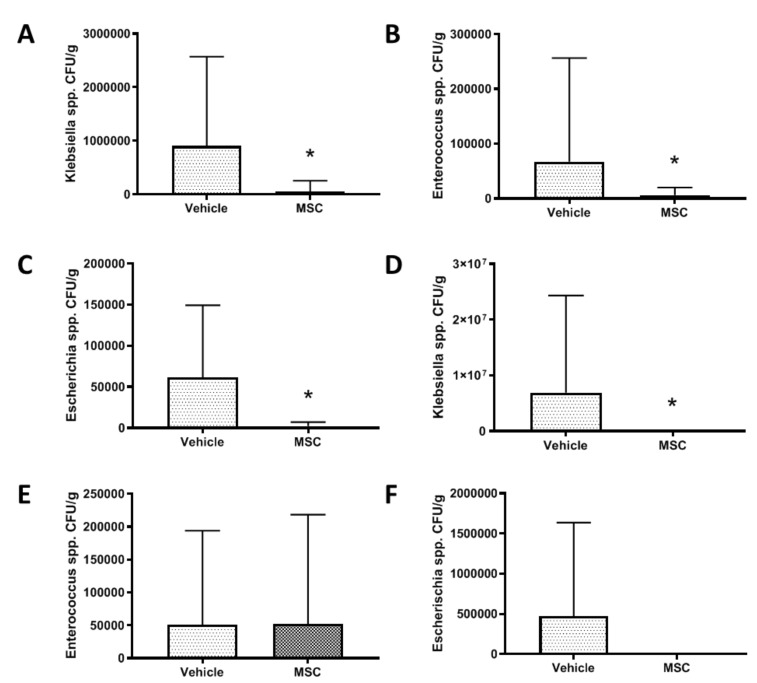
Bacterial CFU reduction after contemporary CD362^+^ UC-MSC administration in severe systemic sepsis. The colony counts representative of *Klebsiella* spp. (**A**), *Enterococcus* spp. (**B**) and *Escherichia* spp. (**C**) were reduced in liver tissue homogenate at 48 h after CD362^+^ UC-MSC administration in animals who underwent severe systemic sepsis. This was also observed in spleen homogenate samples (**D**–**F**). Bars represent mean (*n* = 8/14), error bars represent SD. * = Statistically significant (*p* < 0.05) with respect to control.

Rationale: To determine if the MSC reduces infection levels in various tissues during severe sepsis. Tissue homogenates from animals that completed Series 1 were plated to UTI differential agar plates and the colour of colonies noted. The colony counts representative of *Klebsiella* spp. (Figure 4A), *Enterococcus* spp. (Figure 4B) and *Escherichia* spp. (Figure 4C) were reduced in liver tissue homogenate after CD362^+^ UC-MSC administration compared to vehicle control. This amelioration was also observed in spleen homogenate samples, but to a lesser extent (Figure 4D–F).

### 2.5. CD362^+^ UC-MSCs Administered 4 h Post CLP Reduce Systemic and Organ Presence of Inflammatory Cytokines

**Figure 5:**
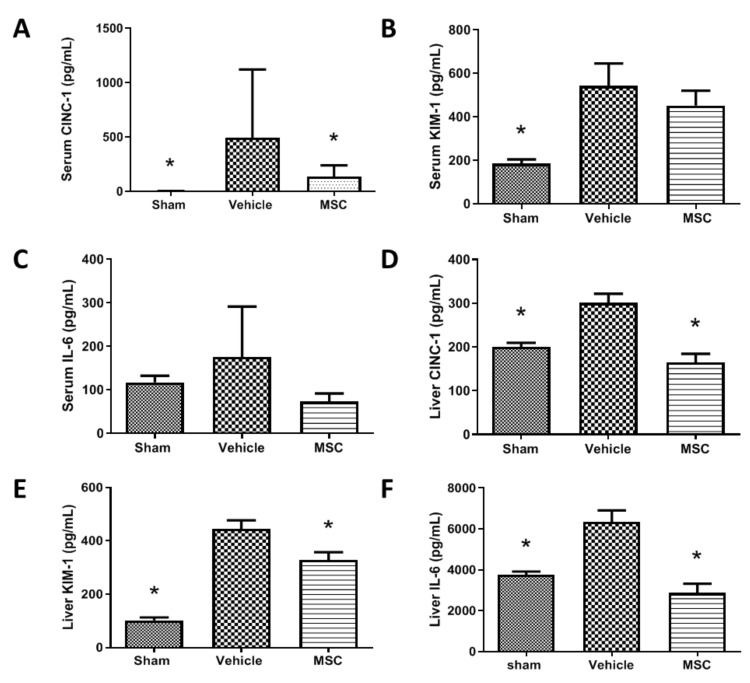
Systemic and organ inflammatory cytokine production after CD362^+^ UC-MSC administration. Serum levels of CINC-1 (**A**), KIM-1 (**B**) and IL-6 (**C**) are reduced in the CD362^+^ UC-MSC treated group compared with vehicle administration. In the liver, levels of CINC-1 (**D**), KIM-1 (**E**) and IL-6 (**F**) are reduced in the CD362^+^ UC-MSC treated group compared with vehicle administration. Bars represent mean (*n* = 3/5/5), error bars represent SD. * Statistically significant (*p* < 0.05).

Rationale: To demonstrate if the MSC delivered at a specific time during the evolution of moderate sepsis can reduce inflammatory markers in serum and tissues. Administration of CD362^+^ UC-MSCs 4 h after performing the CLP procedure reduced the presence of inflammatory cytokines. Animals treated presented lower levels of serum CINC-1 (Figure 5A), KIM-1 (Figure 5B) and IL-6 (Figure 5C) compared with the vehicle control group. This reduction is also observable in liver, where CINC-1 (Figure 5D), KIM-1 (Figure 5E) and IL-6 (Figure 5F) were significantly reduced in the CD362^+^ UC-MSC treated group compared with the vehicle control.

### 2.6. CD362^+^ UC-hMSCs Reduce Systemic Presence of Other Injury-Associated Molecules

**Figure 6:**
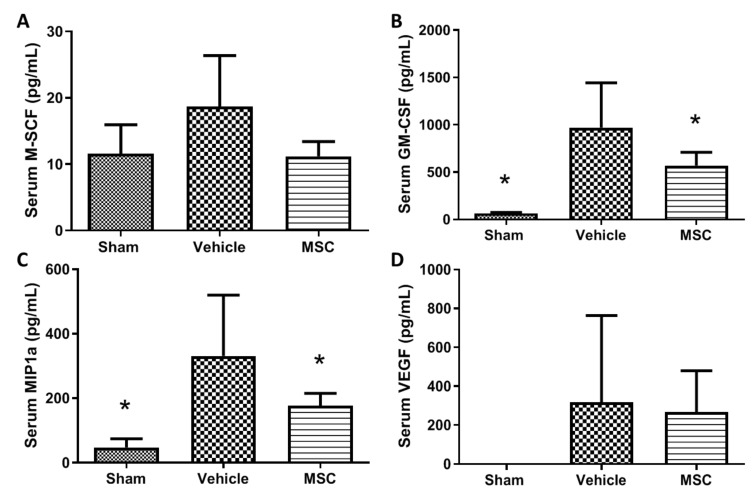
Other systemic soluble factor expression after CD362^+^ UC-MSC administration. Levels of circulating macrophage colony-stimulator factor (M-CSF) (**A**), granulocyte-macrophage colony-stimulating factor (GM-CSF) (**B**), MIP-1α (**C**) and vascular endothelial growth factor (VEGF) (**D**) in serum were reduced in the CD362^+^ UC-MSC treated group compared with vehicle administration. Bars represent mean (*n* = 3/8/8), error bars represent SD. * Statistically significant (*p* < 0.05) with respect to vehicle control group.

Rationale: To demonstrate if the MSC delivered at a specific time during the evolution of moderate sepsis can reduce inflammatory markers in serum and tissues. Besides the clearance of inflammatory cytokines, there is a reduction in the presence of other molecules involved in the inflammatory process after administration of CD362^+^ UC-hMSCs. Treated animals present lower levels of circulating macrophage colony-stimulator factor (M-CSF) (Figure 6A) and granulocyte-macrophage colony-stimulating factor (GM-CSF) (Figure 6B) compared with vehicle control group. These molecules are related with thrombocytopenia in sepsis patients and increased neutrophil infiltration [18,19]. There is also a reduction of the chemoattractant MIP1α (also named CCL3) in the hMSC-treated animals compared with the vehicle control group (Figure 6C). Increased serum levels of vascular endothelial growth factor (VEGF) have been reported in septic patients where it is associated with poor prognosis [20,21]. CD362^+^ UC-hMSC treated animals present lower serum levels of VEGF compared with the vehicle control group (Figure 6D).

## 3. Discussion

Sepsis remains one of the most common causes of death of patients accepted into the ICU [22]. Despite improvements in the management of the infection and organ damage, there is still no specific treatment for sepsis. MSCs have been postulated as a potential treatment for sepsis due to their immune modulation capacity, and in the last years this has been proven in preclinical models [23,24,25,26]. Regardless of the promising performance of MSCs is those experiments, there are still several problems to solve in order to translate these cells to the clinic. One of those problems is the variation between MSC populations, sources, donors etc. [13]. In this study we show that the use of positively selected CD362^+^ umbilical-cord-derived MSCs decreases the severity of CLP-induced sepsis and might be a potential treatment for this affliction in the clinic. We demonstrate that these CD362^+^ UC-MSCs reduce the circulating lactate when administered 4 h post sepsis induction and increase survival in a more severe sepsis injury. The administration of these cells reduces the bacterial load in serum, peritoneal lavage and liver and increases the levels of antibacterial peptides in serum and peritoneal lavage. In addition, animals treated with CD362^+^ UC-MSCs present lower levels of inflammatory cytokines in serum, peritoneal lavage and organs. These data are evidence of the therapeutic potential of a more homogeneous and stable UC-derived CD362^+^ MSC subpopulation for the treatment of polymicrobial systemic sepsis.

Of some additional note here is the utilisation of cryofrozen CD362^+^ UC-MSCs throughout this study, also of an allogeneic (xenogeneic) source. While initially investigators in the field focussed on autologous cell therapy due to safety concerns, allogeneic therapy is rapidly gaining prominence and is widely accepted by regulatory bodies. This is particularly important when it is noted that sick people (particularly geriatric patients with chronic conditions) produce “sick” MSCs, with diminished expansion and therapeutic potential. Furthermore, isolation, production and delivery of large doses of autologous MSCs is not technically feasible in the context of acute, rapid-onset disease such as sepsis.

### 3.1. CD362^+^ UC-MSC-CM Immune Modulation In Vitro

One of the main characteristics that postulate MSCs as a potential therapy for sepsis is their capacity to modulate the immune response in order to control the pro-inflammatory environment found in sepsis patients. CD362^+^ UC-MSC-CM reduced the activation of the NF-κB inflammatory pathway in lung cells after exposure to pro-inflammatory cytokines (cytomix) commonly found to be elevated during the hyperinflammatory phase of sepsis [27]. This CM also had the ability to accelerate the rate of wound closure in the same lung epithelial cells. CD362^+^ UC-MSC-CM also protected gut epithelial and kidney tubule cell lines from pro-inflammatory cytokines, preserving the viability of those cells. Macrophages play a key role in the evolution of the immune response after pathogen infection [28]. In a sepsis state, macrophages produce pro-inflammatory cytokines that lead to dysregulation of the pro/anti-inflammatory balance leading to organ damage [3]. CD362^+^ UC-MSC-CM reduced the production of the pro-inflammatory cytokine IL-6 by peritoneal macrophages exposed to pro-inflammatory stimuli. Another key function of the macrophage is the removal of pathogens through phagocytosis. This process was significantly enhanced through incubation of macrophages with CM, pointing to a possible mechanism to explain the reduced bacterial load seen in sepsis models after MSC administration. In summary, we demonstrated that CD362^+^ UC-MSCs maintain the immune modulation characteristics, previously demonstrated using plastic-adherent MSCs, despite the novel selection method of isolation.

### 3.2. CD362^+^ UC-MSCs Ameliorated CLP-Induced Sepsis

In a more severe systemic sepsis model, mean survival time and total survival at 48 h was improved. However, mortality experiments cannot be used for mechanism of action studies, so further experimental series employed a less severe CLP protocol. Serum lactate is a gold-standard marker for sepsis whereby increased levels of lactate in sepsis patients is correlated with poor prognosis [29]. CD362^+^ UC-MSCs reduced the lactate levels in CLP animals compared with the nontreated group when cells where delivered 4 h after sepsis induction. No effect was observed when CD362^+^ UC-MSCs where administrated at 0 h or 24 h suggesting that the timing of administration plays a key role in the effect of MSCs in the management of sepsis. Administration of CD362^+^ UC-MSCs at the same time as the CLP induction may result in the clearance of the MSCs before the pro-inflammatory environment is established and the cells can exert their action. Administration 24 h after sepsis induction failed to reduce lactate, bacteria or cytokines in our sepsis model—probably by this time the inflammatory process was too advanced to observe the MSCs’ effect. It is therefore likely that with administration at 4 h there was still an appreciable number of viable MSCs circulating which could then be influenced by increasing cytokine and/or bacterial signals to respond through production of the paracrine factors that ultimately confer their therapeutic effect. CD362^+^ UC-MSCs reduced the bacterial load from serum, peritoneal cavity and liver, maintaining the already suggested antibacterial capacity of MSC [17,30,31]. One of the potential mechanisms used by CD362^+^ UC-MSCs to reduce the bacterial load is the increase of antibacterial peptides, such as hepcidin, in serum and the peritoneal cavity compared to nontreated animals. Whether these are predominantly derived from the MSC or the result of stimulated endogenous production remains to be determined with species-specific ELISAs. As mentioned before, the over production of pro-inflammatory cytokines due to pathogen infections results in organ damage and could lead to organ failure and death of the patient. The reduction of inflammatory cytokines in bronchiolar lavage and serum after MSC administration has been documented in several papers over the last years in pneumonia and sepsis models [10,11,24,25,26] and correlates with improving the outcome of the patient. Recently our group showed that MSCs selected using the CD362 marker maintain this anti-inflammatory activity in an *E. coli* pneumonia model [15] and in a ventilation injury model [14]. Supporting these discoveries, we showed CD362^+^ UC-MSCs reduced the pro-inflammatory cytokines CINC-1 and IL-6 in circulation, peritoneal cavity and liver and also reduced the kidney injury marker KIM-1 in serum, peritoneal lavage and liver. Combining our in vitro and in vivo models, our findings suggest that inflammation can ultimately lead to loss of cell viability in target organs, and the reduction of inflammatory cytokines is one of the possible protective mechanisms of CD362^+^ UC-MSCs in sepsis.

## 4. Materials and Methods

All work was approved by the Animal Care in Research Ethics Committee of the National University of Ireland, Galway, and conducted under license from the Health Products Regulatory Agency, Ireland (AE19125/P045). Specific pathogen-free adult male Sprague Dawley rats (Charles River Laboratories, Kent, UK) weighing between 300 and 450 g were used in all experiments.

### 4.1. CD362-Targeted Isolation from Human Umbilical Cord Tissue

Human umbilical cord (hUC)-derived CD362^+^ MSC cell populations were provided by Orbsen Therapeutics Ltd. (Galway, Ireland). CD362^+^ UC-hMSC were prepared by a protocol similar to human bone marrow CD362^+^ MSC as previously described [14]. All hMSC populations were cultured at 37 °C, 95% humidity, 5% CO_2_ and hypoxic conditions of 2% O_2_ until 70–80% confluent, and then trypsinised (Gibco; Biosciences, Dublin, Ireland), and culture expanded to passage 3–4, whereupon they were trypsinised, resuspended and cryopreserved. Each cryovial containing 1 × 10^7^ cryopreserved cells in 1 mL was quickly thawed with 9 mL of PBS. Trypan blue (Sigma Aldrich Ltd., Wicklow, Ireland) exclusion dye staining was performed immediately post-thaw, indicating viability of 94.3 ± 1.5%. The required target dose, one million MSCs for each 100 g of animal, was pelleted at 400× *g* for 5 min and resuspended in 1 mL of PBS ready for administration. Of note, CD362 levels rapidly diminish with passaging post isolation, and at the time of administration, all cell populations had identical low expression (data not shown).

### 4.2. Generation of Conditioned Medium

MSCs were seeded at 10,000/cm^2^ in T175 flasks (Greiner; Cruinn Diagnostics Ltd., Dublin, Ireland), and two days later medium was aspirated and replaced with serum-free MEM-α for a further 24 h. In all experiments, medium was completely replaced by MSC-conditioned medium or vehicle. Control (vehicle) was unconditioned MEM-α.

### 4.3. In Vitro Determination of the Effects of CD362^+^ Human Mesenchymal Stromal Cells

- Nuclear Factor κB activation assay. A cell line derivative of type II alveolar A549 cells incorporating a stably transfected κB-luciferase reporter construct (Thermo Fisher, Waltham, MA, USA) was grown to confluence. Cell monolayers were randomised to receive cytomix (interleukin 1β (IL-1β) (10 ng/mL), TNF-α (50 ng/mL) and IFN-γ (50 ng/mL) (Immunotools Ltd., Friesoythe, Germany), or sham (vehicle) injury, then treated with either conditioned medium (CM) from CD362^+^ UC-MSCs, or vehicle. Cells were harvested at 24 h and assayed for luciferase content as an indicator of NF-κB activation.
- Scratch wound assay. A549 lung epithelial cells were seeded to 24-well plates at 100,000 cells per cm^2^ and the following day a single scratch wound was introduced per well with a p1000 pipette tip. Wells were aspirated, rinsed with PBS and re-fed with CD362^+^ UC-MSC-CM or vehicle. At various timepoints up to 24 h, scratch wounds were imaged by light microscopy and wound width assessed through measurement of pixel distance across the wound.
- Cell viability. 3-(4, 5-dimethylthiazol-2-yl)-2,5-diphenyltetrazolium bromide (MTT) assay was performed using MTT reagent (thiazolyl blue tetrazolium bromide; Sigma Aldrich Ltd.) reconstituted in culture medium (5 mg/mL) to evaluate cell viability and proliferation. Human colonic adenocarcinoma cell line (T84) and human kidney proximal tubular cell line (HK-2) monolayers were randomised to receive cytomix, or sham (vehicle) activation, then treated with either CD362^+^ UC-MSC-CM, or vehicle. After treatment, cells were washed with PBS, followed by incubation with MTT reagent for 3 h at 37 °C in a humidified cell culture incubator. Cell supernatant was replaced with dimethyl sulfoxide and absorbance readings were measured using the Varioskan™ Flash microplate reader (Thermo Fisher Ltd.) at 595 nm wavelength. The degree of cell viability was presented as a percentage relative to uninjured control.
- Inflammatory cytokines production. Peritoneal macrophages were isolated from healthy rats by performing a peritoneal lavage with 20 mL of PBS, seeded to 96-well plates (c. 90,000 per well), and randomised to receive 100 ng/mL or 250 ng/mL of *E. coli* lipopolysaccharide (LPS), or sham (vehicle) activation, then treated with either CD362^+^ UC-MSC-CM, or vehicle. Interleukin 6—a cytokine under tight control of the NF-κB promoter [32]—production was measured by ELISA (R&D Systems, UK).
- Phagocytosis assay. THP-1 monocytes were seeded at 100,000 cells per cm^2^ in 96-well plates and exposed to PMA (1 µg/mL) for 72 h to force differentiation to macrophages. Lipopolysaccharide (100 or 250 ng/mL) was added for a further 24 h. Zymosan particles were then added for 4 h and phagocytosis quantified through counting of engulfed spots inside macrophages under fluorescent microscopy. Two fluorescent spots were considered positive for phagocytosis.

### 4.4. In Vivo Experimental Protocols

- CLP-induced sepsis. Adult male Sprague Dawley rats were anaesthetised by isoflurane inhalation. In order to induce polymicrobial sepsis, a midline laparotomy was performed, the caecum was exteriorised, and a 4–0 silk ligature was placed 5 mm from the caecal tip. The caecum was punctured twice with a biopsy punch (2 mm diameter for severe and 1 mm diameter for moderate severity) and a small amount of faecal content was extruded. The caecum was returned to the abdominal cavity and the midline incision was sutured. The rats were monitored and their status, including behavioural signs and welfare, was recorded up to 48 h after the CLP procedure.

### 4.5. Caecal Ligation and Puncture Systemic Sepsis Model

- Experimental series. Series 1: MSC administered within 30 min after severe CLP surgery (0 h). Groups: sham surgery (4), vehicle (9), CD362^+^ UC-MSC (10). Series 2: MSC administered 24 h after moderate CLP surgery (24 h). Groups: sham surgery (4), vehicle (9), CD362^+^ UC-MSC (11). Series 3: MSC administered 4 h after moderate CLP surgery (4 h). Groups: sham surgery (5), vehicle (12), CD362^+^ UC-MSC (10).
- Survival. In Series 1, animals were euthanised if they reached humane endpoints and time recorded. All animals were euthanised at 48 h.
- In Vivo assessment. At 48 h post CLP sepsis induction, animals were anaesthetised with intraperitoneal ketamine (80 mg.kg^−1^, Ketalar™; Pfizer, Cork, Ireland) and medetomidine (0.5 mg.kg^-1^, Dormidor™, Vetoquinol Ltd., Buckingham, UK). After confirmation of depth of anaesthesia by paw clamp, IV access was obtained via tail vein. Surgical tracheostomy was performed, using a 12G tracheostomy tube. Following intra-arterial access for blood sample analysis and monitoring, anaesthesia was maintained with alfaxalone (2 mg.kg^-1^, Alfaxan™; Vetoquinol Ltd.) and paralysis with cisatracurium besylate (0.5 mg.kg^-1^, Tracrium™; GlaxoSmithKline PLC., London, UK) and mechanical ventilation was commenced. Arterial blood lactate analysis was performed as previously described [10].
- Ex Vivo assessment. After exsanguination under anaesthesia, bronchoalveolar lavage (BAL) and peritoneal lavage (PL) were performed. Tissue samples from lung, liver, kidney and heart were collected for cytokine profiles and bacterial load measurements.
- Bacterial load. From the severe sepsis series, liver and spleen were immediately rinsed in 70% isopropyl alcohol and homogenised in 10 mL of PBS per gram of tissue. Homogenate was serial-diluted in PBS, plated to UTI agar plates (Fannin Ltd., Galway, Ireland) and incubated overnight at 37 °C. Total colony numbers of each indicative colour were counted. In the 4 h therapeutic moderate sepsis series, total DNA was isolated from serum, peritoneal lavage and organs by homogenisation and DNA affinity column (DNeasy Blood and Tissue Kit; Qiagen Ltd., Manchester, UK). PCR was performed using the following set of primers: forward 5′-TCCTACGGGAGGCAGCAGT-3′ (Tm 59.4 °C), reverse, 5′-GGACTACCAGGGTATCTAATCCTGTT-3′ (Tm 58.1 °C) targeting the 16S fraction of bacterial ribosomal RNA [33].
- Inflammatory cytokine profile. Cytokine-induced neutrophils chemoattractant (CINC-1), kidney injury molecule (KIM-1), interleukin 6 (IL-6) and hepcidin were quantified by ELISA (R&D Systems) and 23 other cytokines and growth factors where measured using a multiplex immunoassay system (Bio-Plex Pro Rat Cytokine, Chemokine and Growth Factor Assay; Bio-Rad Ltd., Watford, UK). The full multiplex dataset is available as Supplemental File S2.
- Statistical analyses. Data were analysed with GraphPad Prism software (GraphPad Software Ltd., San Diego, CA, USA). The distribution of all data was tested for normality with Kolmogorov–Smirnov tests. Data were analysed by two-way or one-way ANOVA or ANOVA on Ranks (Kruskall–Wallis) as appropriate, with post hoc testing by Dunnett ’s method, with the vehicle group as the single comparison group, or with Student–Newman–Keuls between-group comparisons, as appropriate. Underlying model assumptions were deemed appropriate on the basis of suitable residual plots. A two-tailed *p* value of less than 0.05 was considered significant.

## 5. Conclusions

In these studies, we demonstrated therapeutic potential of CD362^+^ UC-MSCs ameliorating sepsis injury in a polymicrobial sepsis model when delivered 4 h after sepsis induction, but not at 0 h and 24 h, suggesting the importance of timing in the administration of MSC therapy. We also demonstrated the immune modulation capacity of CD362^+^ UC-MSCs in vitro and enhanced survival in a more severe model of systemic sepsis. All this taken together suggests that isolation of MSCs using the CD362 surface marker from umbilical cord produces a cell population that maintains the immune modulation properties expected from MSCs and that CD362^+^ UC-MSCs can offer a therapy for sepsis.

## Supplementary Materials

Supplementary materials consist of Supplemental File S1 (additional CLP results) and Supplemental File S2 (complete cytokine multiplex dataset) and can be found at https://www.mdpi.com/1422-0067/21/21/8270/s1.

## Abbreviations

ATMP	Advanced Therapeutic Medicinal Products
BM	Bone Marrow
CLP	Caecal Ligation and Puncture
ICU	Intensive Care Unit
IFN	Interferon
IL	Interleukin
KIM-1	Kidney Injury Marker 1
MSC	Mesenchymal Stromal Cell
PBS	Phosphate Buffered Saline
PMA	Phorbol-12-Myristate-13-Acetate
TNF	Tumour Necrosis Factor
UC	Umbilical Cord
UTI	Urinary Tract Infection
