# Supplementary figures and images for: Umbilical Cord-Derived CD362+ Mesenchymal Stromal Cells Attenuate Polymicrobial Sepsis Induced by Caecal Ligation and Puncture
Source: Int J Mol Sci. 2020 Nov 4;21(21):8270. doi: 10.3390/ijms21218270 (PMC7672591; doi:10.3390/ijms21218270)

# HEART

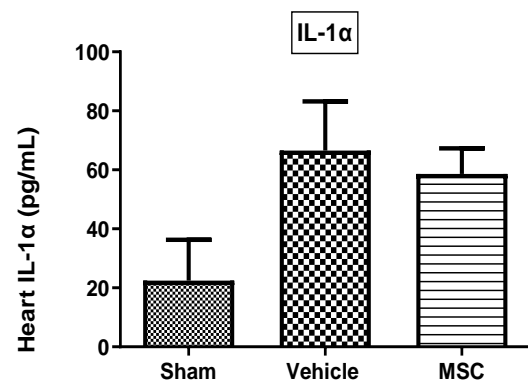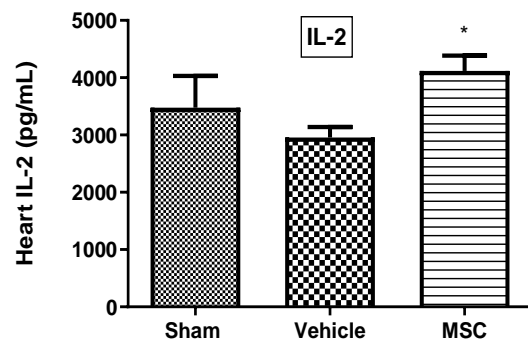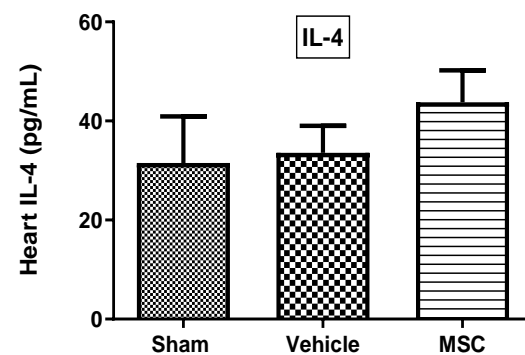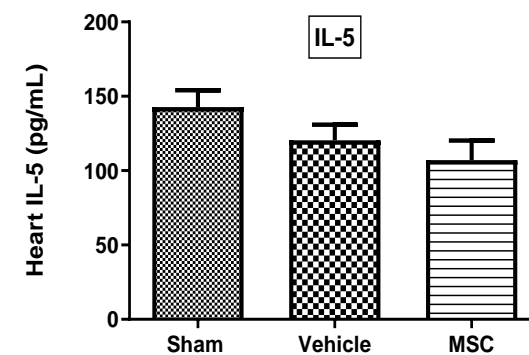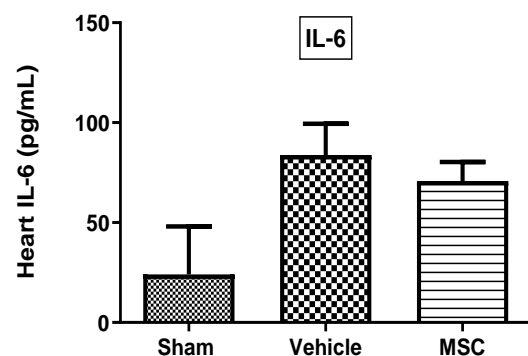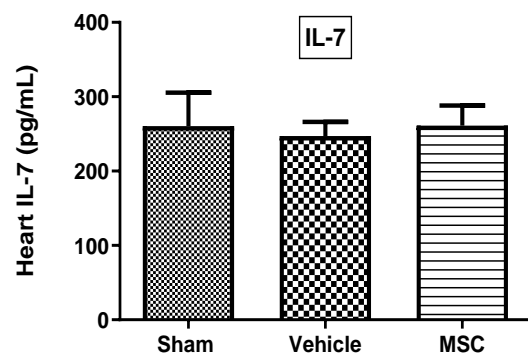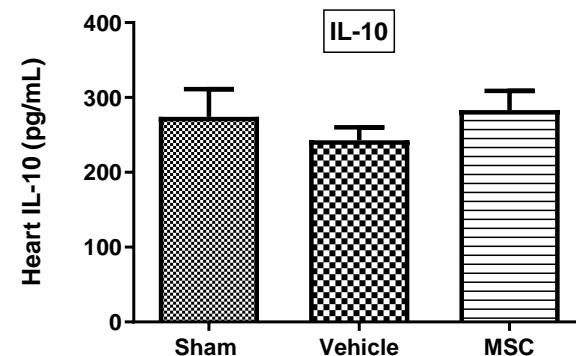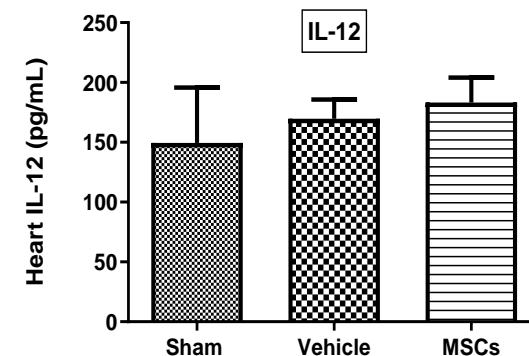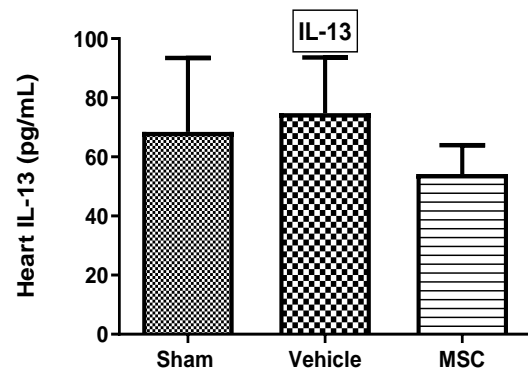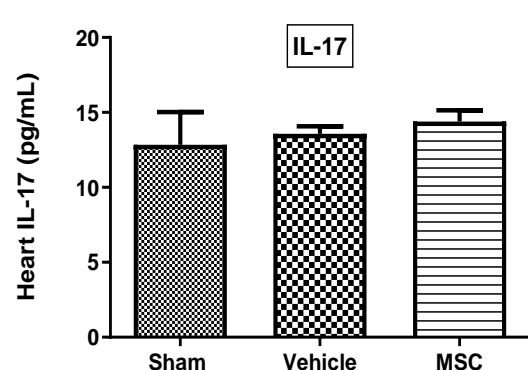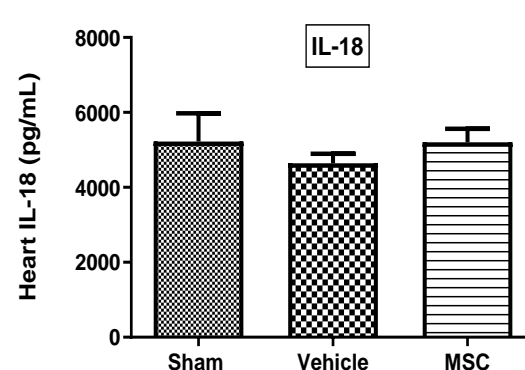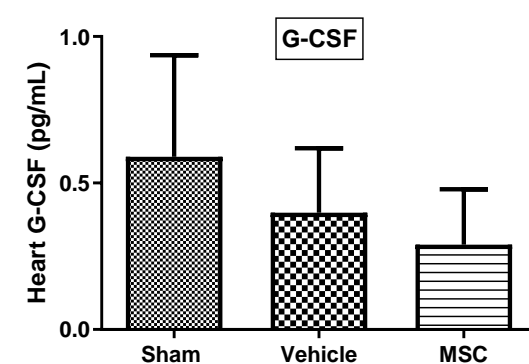

# HEART

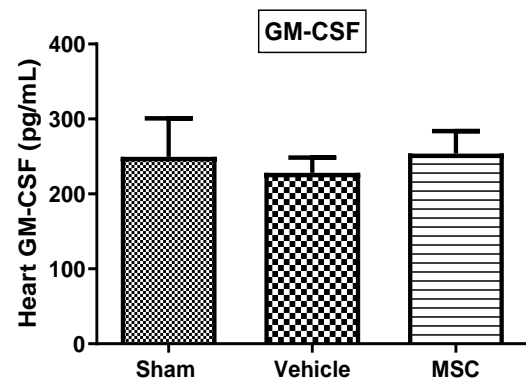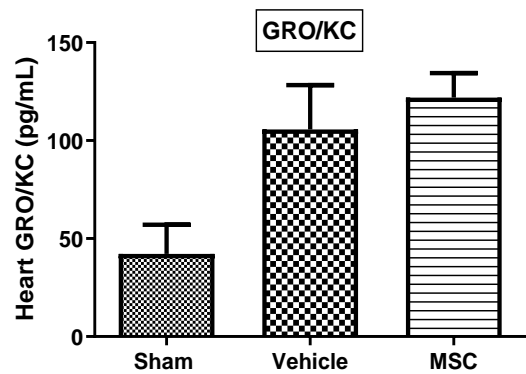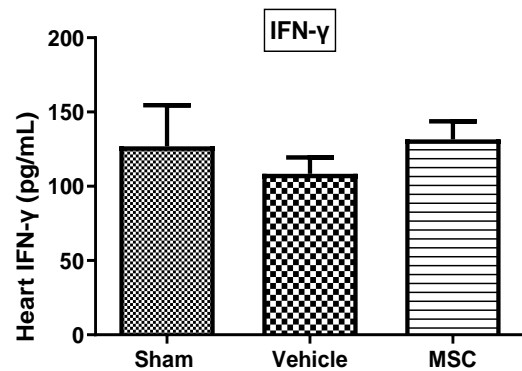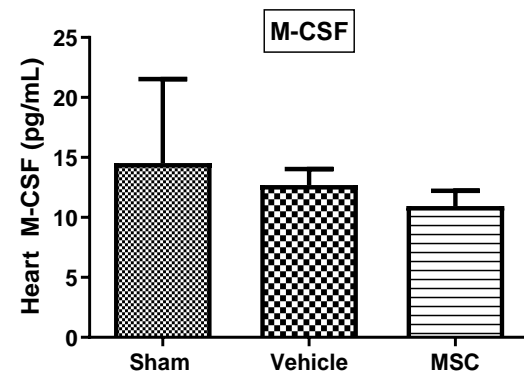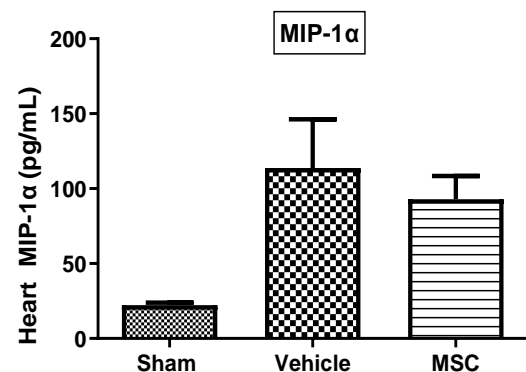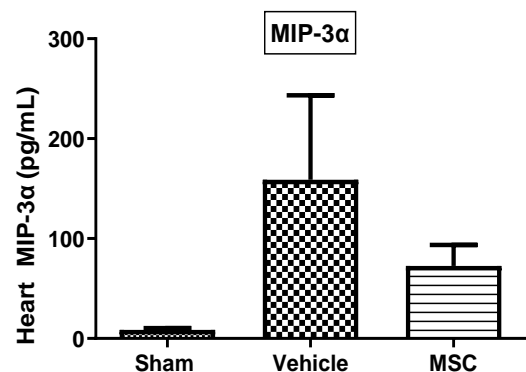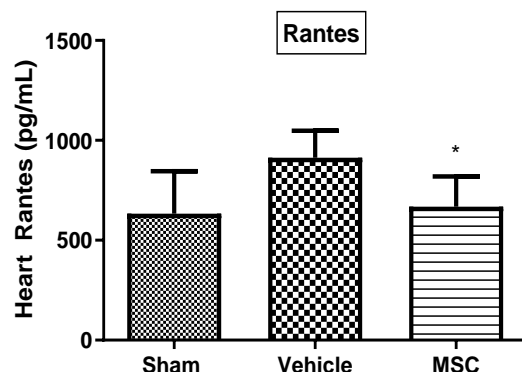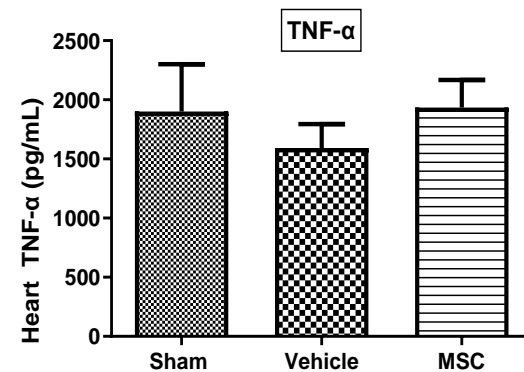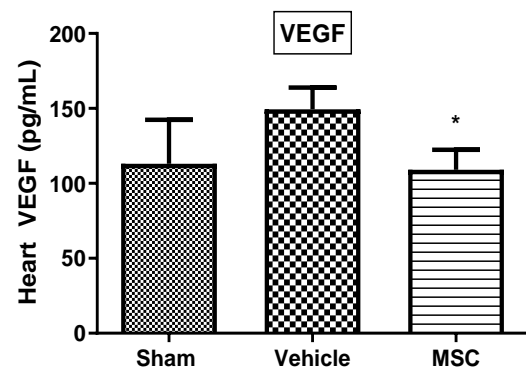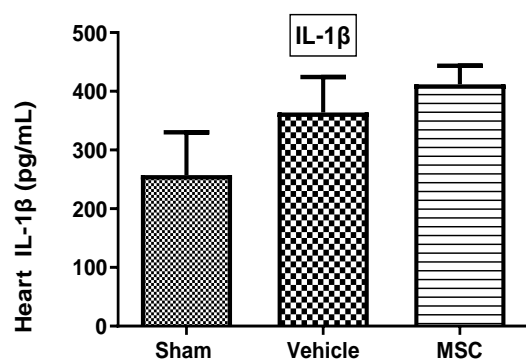

LIVER

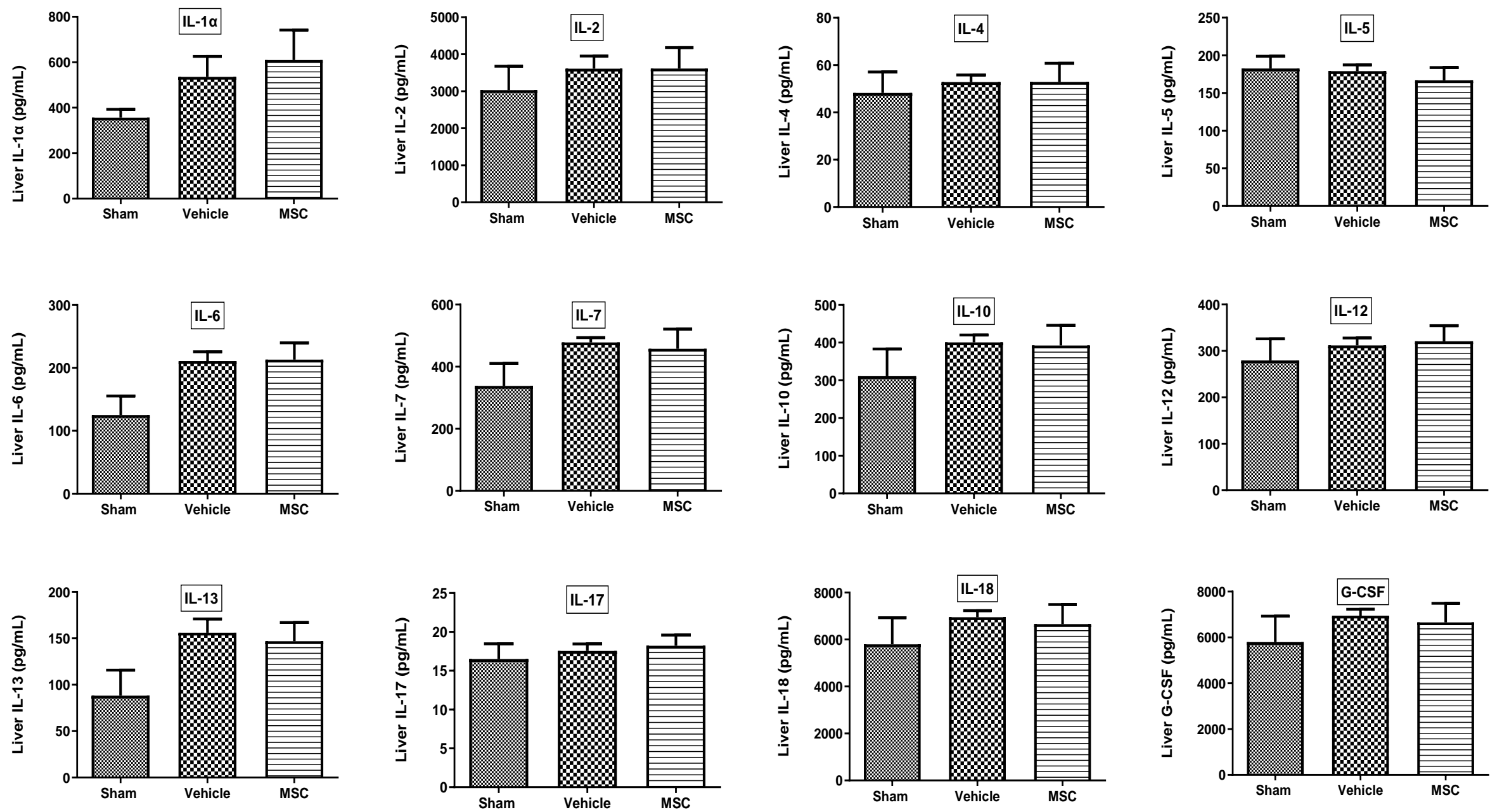

LIVER

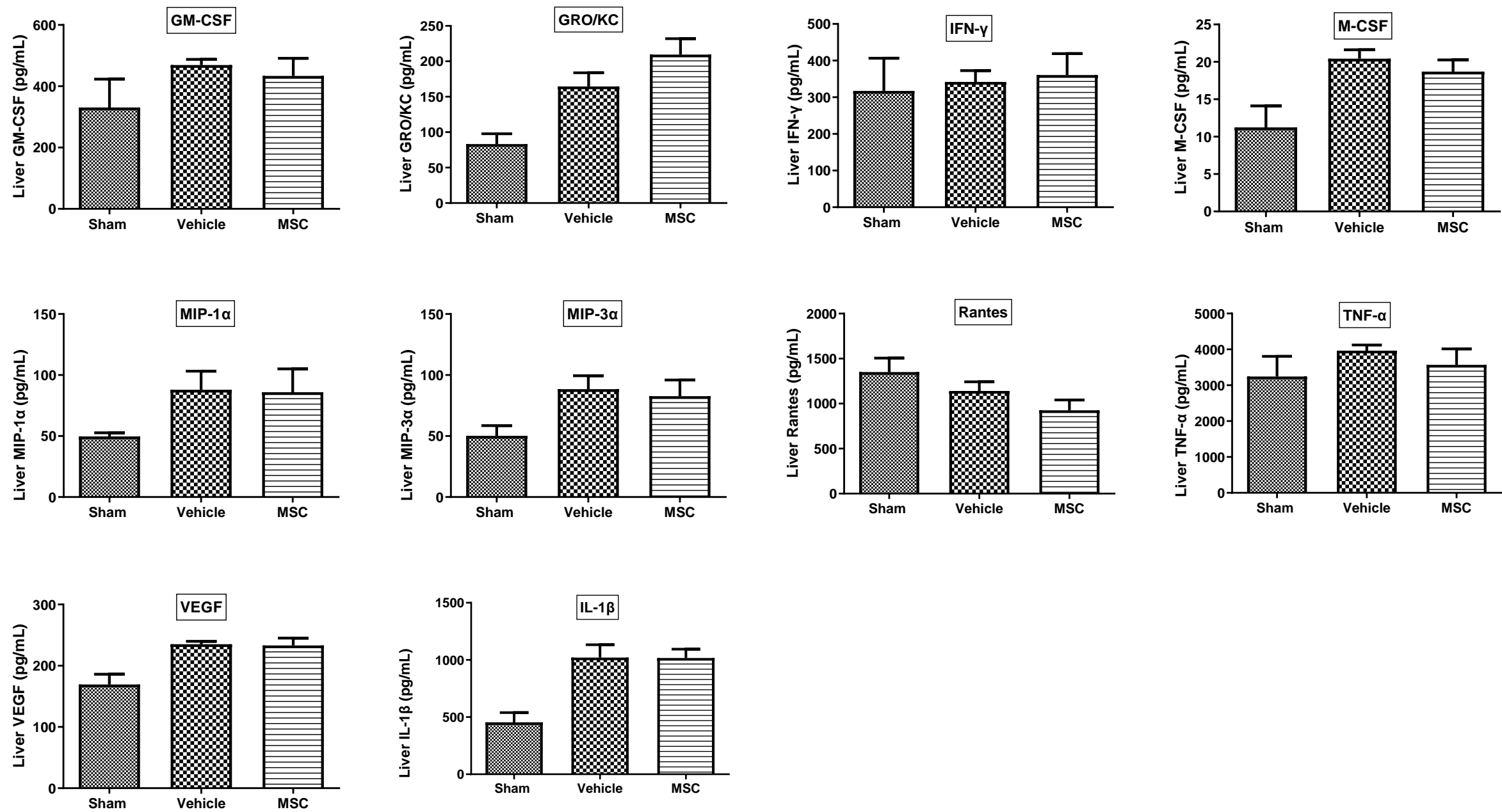

SERUM

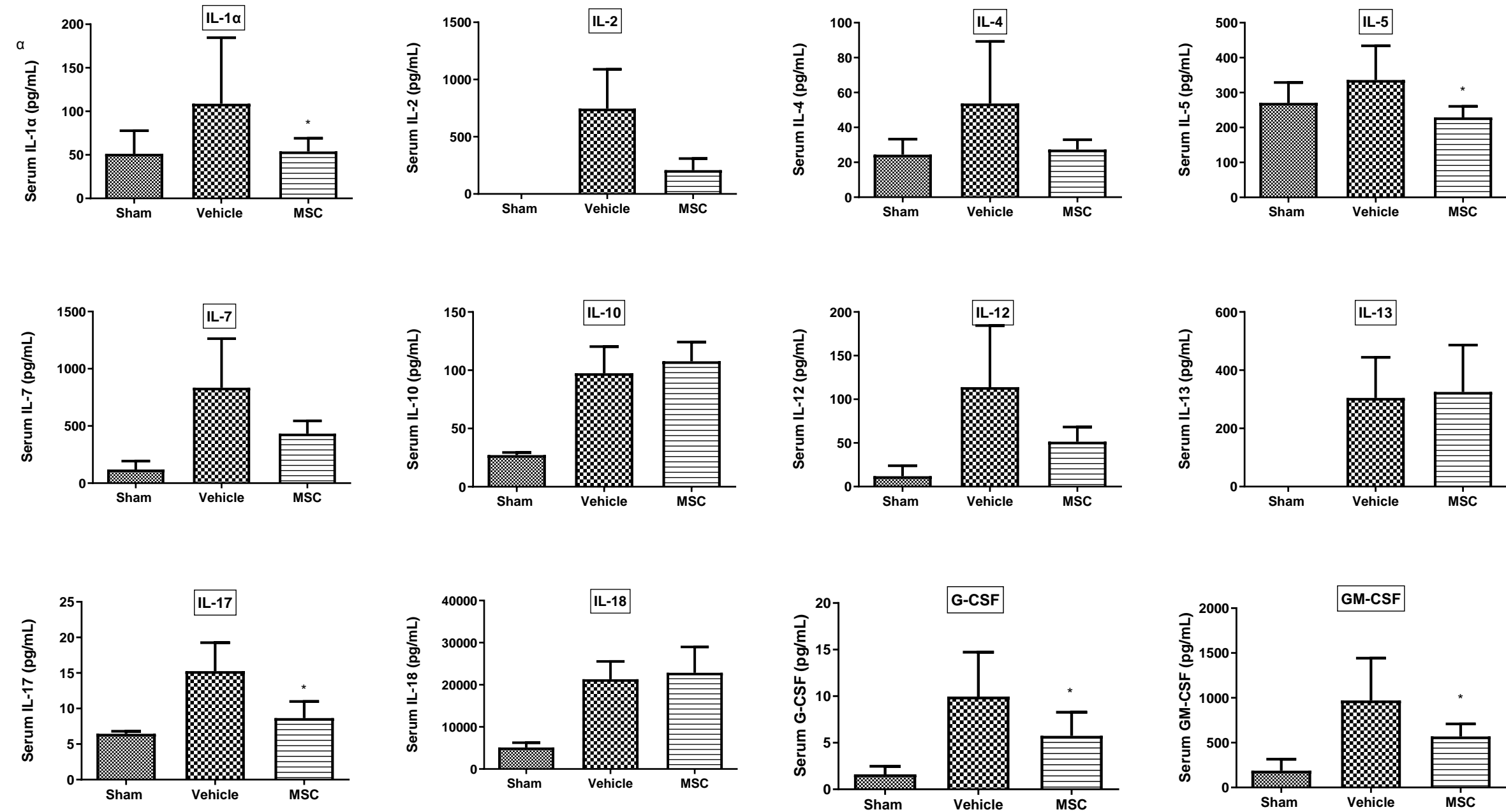

SERUM

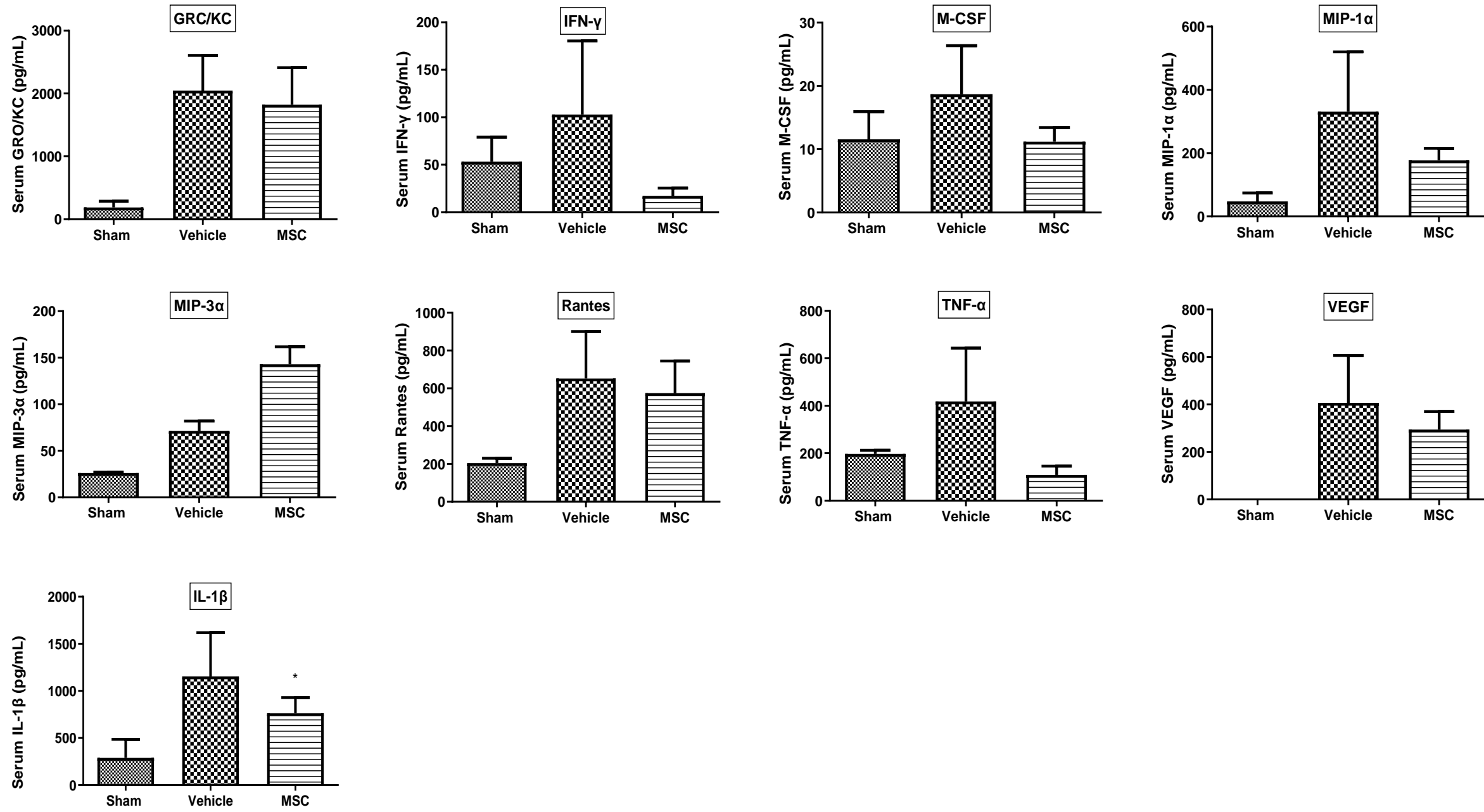

Supplement: Supplementary file 1 [file ijms-21-08270-s001.zip › Supplemental File S2.pdf]
