# Supplementary material for: Umbilical Cord-Derived CD362+ Mesenchymal Stromal Cells Attenuate Polymicrobial Sepsis Induced by Caecal Ligation and Puncture
Source: Int J Mol Sci. 2020 Nov 4;21(21):8270. doi: 10.3390/ijms21218270 (PMC7672591; doi:10.3390/ijms21218270)
Supplement: Supplementary file 1 [file ijms-21-08270-s001.zip › Supplemental File S1.doc]

**Figure S1. CD362^+^ UC-MSC administration contemporary with CLP or at 24 hours delayed does not affect serum lactate or KIM-1 levels.** Administration of CD362^+^ UC-MSCs either contemporaneously or 4 hours after CLP induction did not significantly affect lactate measured in serum 48 hours after the CLP procedure compared with vehicle (Panel A). Administration at either time point also failed to alter Kidney Injury Marker-1 levels in seruml (Panel B). Bars represent mean (n=3//8/8/8), error bars represent SD. * Statistically significant (P < 0.05) with respect to vehicle group.
